# Detectability assessment of a satellite sensor for lower tropospheric ozone responses to its precursors emission changes in East Asian summer

**DOI:** 10.1038/s41598-019-55759-7

**Published:** 2019-12-23

**Authors:** Mizuo Kajino, Sachiko Hayashida, Tsuyoshi Thomas Sekiyama, Makoto Deushi, Kazuki Ito, Xiong Liu

**Affiliations:** 1 Meteorological Research Institute (MRI), Japan Meteorological Agency (JMA), Tsukuba, Ibaraki, 305-0052 Japan; 2 Faculty of Life and Environmental Sciences, University of Tsukuba, Tsukuba, Ibaraki, 305-8572 Japan; 3 Faculty of Science, Nara Women’s University, Nara, 630-8506 Japan; 4 Graduate School of Life and Environmental Sciences, University of Tsukuba, Tsukuba, Ibaraki, 305-8572 Japan; 5 Harvard-Smithsonian Center for Astrophysics, Cambridge, MA 02138 USA

**Keywords:** Atmospheric chemistry, Environmental impact

## Abstract

Satellite sensors are powerful tools to monitor the spatiotemporal variations of air pollutants in large scales, but it has been challenging to detect surface O_3_ due to the presence of abundant stratospheric and upper tropospheric O_3_. East Asia is one of the most polluted regions in the world, but anthropogenic emissions such as NO_x_ and SO_2_ began to decrease in 2010s. This trend was well observed by satellites, but the spatiotemporal impacts of these emission trends on O_3_ have not been well understood. Recent advancement in a retrieval method for the Ozone Monitoring Instrument (OMI) sensor enabled detection of lower tropospheric O_3_ and its legitimacy has been validated. In this study, we investigated the statistical significance for the OMI sensor to detect the lower tropospheric O_3_ responses to the future emission reduction of the O_3_ precursor gases over East Asia in summer, by utilizing a regional chemistry model. The emission reduction of 10, 25, 50, and 90% resulted in 4.4, 11, 23, and 53% decrease of the areal and monthly mean daytime simulated satellite-detectable O_3_ (ΔO_3_), respectively. The fractions of significant areas are 55, 84, 93, and 96% at a one-sided 95% confidence interval. Because of the recent advancement of satellite sensor technologies (e.g., TROPOMI), study on tropospheric photochemistry will be rapidly advanced in the near future. The current study proved the usefulness of such satellite analyses on the lower tropospheric O_3_ and its perturbations due to the precursor gas emission controls.

## Introduction

Surface O_3_ causes detrimental effects on plants and animals^1^. Tropospheric O_3_ is an important climate forcing agent causing global warming^2^. Surface and lower tropospheric O_3_ is mainly produced by photochemical reactions between NO_x_ and non-methane volatile organic compounds (NMVOCs). The Asian anthropogenic nitrogen oxide emissions surpassed those from North America and Europe in 1990s^3^ and since then the emissions continued to increase in 2000s^4^. Cooper *et al*.^5^ showed based on the *in-situ* measurements that the daytime O_3_ in the summer in China was remarkably larger than that over other locations in the world in 2000s. In 2010s, the Chinese anthropogenic emissions such as NO_x_ and SO_2_ began to decrease and this trend was well observed by satellites^6–9^. However, because the reduction of NO_x_ emissions increased photochemical production of O_3_ near the emission source regions (VOC-limited regions)^10^, it is not necessary that the lower tropospheric O_3_ in China and East Asia have started to decrease, associated with the decrease in NO_x_ emission in China, Korea, and Japan. Also the local/regional emission reduction may not decrease local/regional O_3_ concentrations, because the trans-boundary to hemispheric transport contributes to raise the background level of the surface and the lower tropospheric O_3_ concentrations^11^.

In order to monitor the spatiotemporal variations of air pollutants in large scales and to detect their long-term trends, satellite sensors are powerful tools. However, in terms of the surface and lower tropospheric O_3_, it has been challenging to measure from the space, because of the presence of abundant stratospheric O_3_. Recently, a retrieval algorithm has been developed by Liu *et al*.^12^ to detect the lower-tropospheric (i.e., approximately 0–3 km) O_3_ and its legitimacy has been validated especially focusing on the East Asian region^13–16^. In fact, based on the climatological zonal and monthly mean O_3_ profiles derived from 15 years of ozonesonde measurements^17^, the 0–3 km column amounts in the northern mid-latitudes range from 7.1 to 13.7 DU, which are only 2.1–4.0% of the total column amounts, approximately 300–380 DU. In this study, we investigated the statistical significance for the OMI sensor to detect the tropospheric O_3_ responses to the current/future emission reductions of the O_3_ precursor gases (NO_x_ and NMVOCs) over East Asia by using the retrieval algorithm and numerical simulations. We selected June 2006 in the study, because both observed and simulated lower tropospheric O_3_ concentrations were largest. Also, the summer was most favorable for the current analysis, evaluating sensitivity of O_3_ to local precursor emission changes. In colder seasons, the contribution of long-range transport is larger due to monsoon and lower photochemical production. More than half of surface O_3_ in East Asia were attributed to those transported from distant sources, whereas most of them were the domestic origin in the summer^11^. In addition, the retrieval sensitivity to lower tropospheric O_3_ in this region maximizes in the summer due to smaller solar zenith angle^12^.

## Results and Discussion

### Spatial distribution of lower tropospheric O3

**Figure 1:**
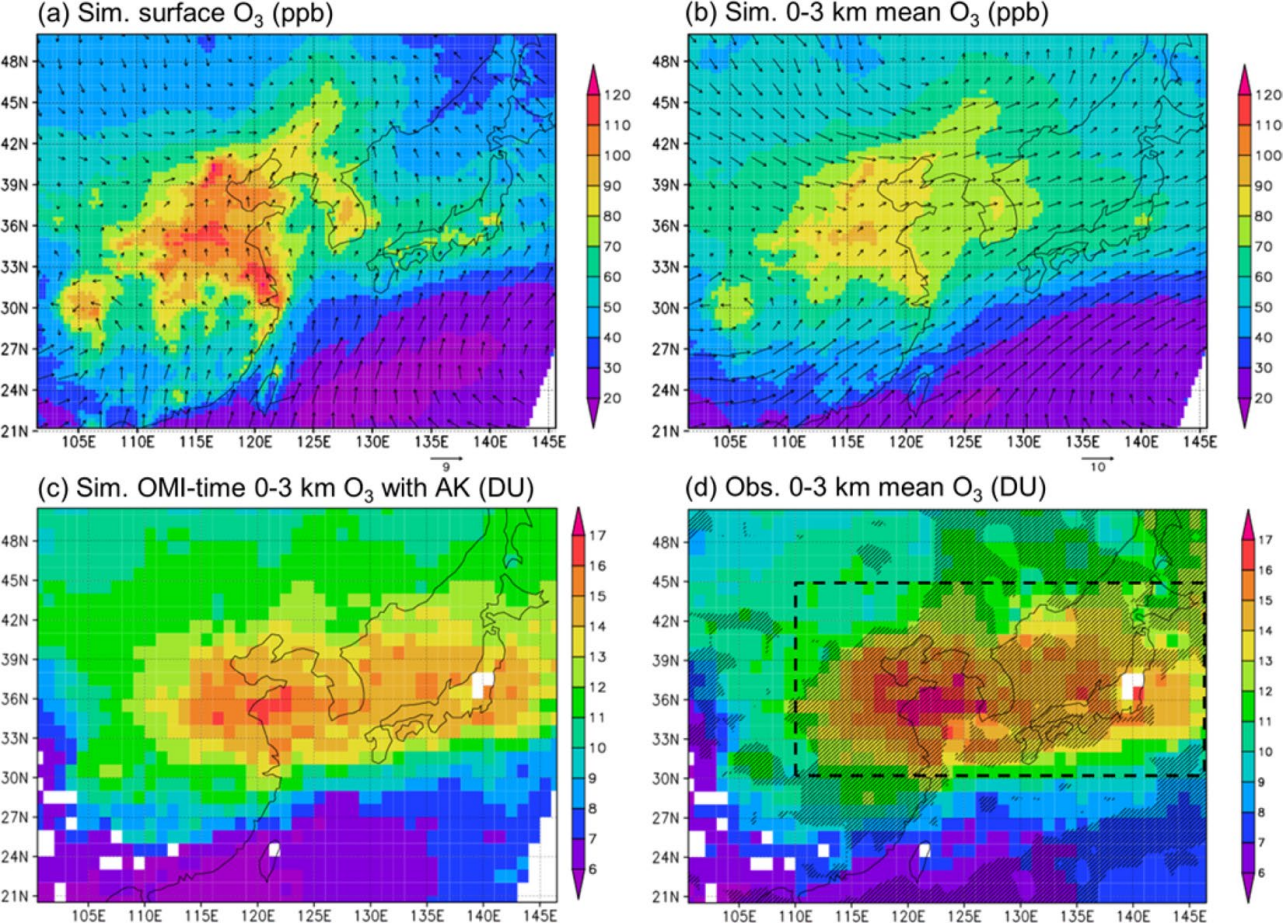
Monthly mean simulated and observed lower tropospheric O_3_. (**a,b**), The monthly mean simulated surface and 0–3 km mean O_3_ mixing ratio (ppb) together with wind vectors in June 2006. **c**, The monthly mean simulated OMI-time 0–3 km O_3_ column amount (DU) with AK and **d**, OMI-observed 0–3 km O_3_ column amount (DU). The dashed box indicates the analysis area of the study (110–146°E, 30–45°N) and the hatched area indicates the grids where the retrieved OMI-O_3_ is significantly larger than the a priori (climatological) O_3_ at a one-sided 95% confidence interval. The spatial resolution Δ*x* of (**a,b**) is 30 km, whereas Δ*x* of (**c,d**) is 1 degree.

Figure 1 shows the monthly mean simulated and observed O_3_ over East Asia in June 2006. The simulation was conducted by using a regional meteorology – chemistry model (NHM-Chem)^18^. The panels are to show how the simulation results were compared with the retrieved OMI O_3_^12,19–21^ (denoted as OMI-O_3_ hereinafter) and how the horizontal distributions were changed accordingly by the averaging procedures. Figure 1a shows the simulated surface O_3_ concentration (ppb; Δ*x* = 30 km), the most important factor for the terrestrial ecosystems, and thus to be evaluated by the observation. In the summer, the atmospheric conditions are most favorable for the photochemical production of O_3_ over the land. Because the surface wind was relatively weaker compared to the cold seasons, the surface concentration showed maxima over the high emission areas, such as North China Plain, Yangtze River Delta, and Sichuan Basin in China and densely populated regions in Korea and Japan. In fact, the cluster analysis, provided by Hayashida *et al*.^15^, proved that the locations of a high OMI-O_3_ cluster matched with the high NO_x_ emission areas. In the month, the Pacific High pressure system was dominant and the clean maritime air was transported from the south to the Northwestern Pacific. During the summer, the Pacific High blocks the long-range transport due to the mid-latitude westerlies in the region. Figure 1b shows the simulated 0–3 km mean O_3_ concentration (ppb; Δ*x* = 30 km). Due to the prevailing mid-latitude westerlies at upper altitudes, the high concentration areas extend farther eastward compared to the surface concentrations. Figure 1c shows the simulated OMI-time 0–3 km mean O_3_ column amount (DU; Δ*x* = 1°), convolved with the retrieval averaging kernels (AKs) (denoted as simulated O_3_ with AK, hereinafter) (see Eq. (1)), which can be quantitatively compared with the OMI-O_3_ as shown in Fig. 1d. The “simulated OMI-time” indicates simulation results at 6 UTC (13:40 local time at the center of model domain (115 °E)), while the OMI observation time is 13:45 local time. The spatial distributions of Fig. 1b,c are drastically different due to the following reasons: (1) influences of O_3_ from the above layers and (2) exclusion of cloudy/rainy days and days under the influence of stratospheric O_3_ intrusion due to the tropopause perturbations. In order to clearly show the effects, two more panels (OMI-time O_3_ before and after screening for clouds and stratospheric inclusion) are added to Fig. 1 as illustrated in Fig. S1 in the supplement. The “simulated OMI-time 0–3 km O_3_” means that after the screening in this paper.

The Student’s *t*-test was applied for the monthly mean OMI-O_3_ and the a priori O_3_ using the daily values in June 2006. Degrees of freedom at each grid point are the number of available data in the month minus one, i.e., up to 29. The hatched area of Fig. 1d indicates the grids where the OMI-O_3_ was significantly larger than the a priori O_3_ at a one-sided 95% confidence interval. Most of the O_3_ rich regions (i.e., >12 DU) are statistically significant. For the emission reduction test as presented later, data in the hatched area inside the dashed box (110–146°E, 30–45°N) were used for the analysis.

The same horizontal distribution with a different confidence interval, two-sided 99%, is presented in Fig. S2, in the supplement. Over the relatively low concentration areas such as Sichuan Basin, Korea, and Japan, the hatched areas become small or disappeared. In contrast, over the high concentration areas such as North China Plain and Yangtze River Delta, the difference between the OMI-O_3_ and the a priori O_3_ remained significant at this confidence interval, due to substantially large near-surface photochemical O_3_ production.

### Comparison between observation and simulation

**Figure 2:**
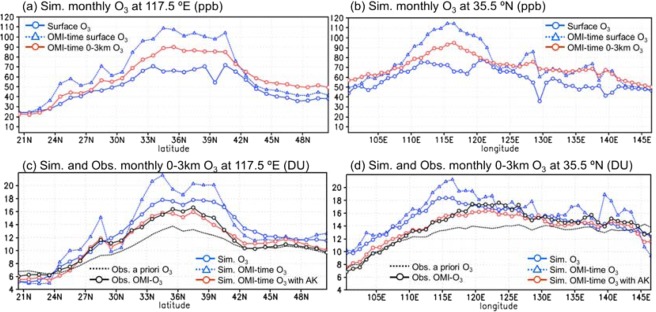
Cross sections of monthly mean simulated and observed lower tropospheric O_3_. The cross sections of monthly mean (**a**,**b)**, surface O_3_ mixing ratio (ppb) and (**c**,**d)**, 0–3 km O_3_ column amount (DU) at (**a**,**c)**, 117.5°E and (**b**,**d)**, 35.5°N. **a**,**b**, The blue solid and dashed lines indicate the monthly mean values of simulated hourly O_3_ and OMI-time O_3_, respectively. The red line indicates the monthly mean OMI-time 0–3 km averaged O_3_ (after the screening). (**c**,**d**) The black solid and dashed lines indicate the retrieved OMI-O_3_ and the a priori O_3_ used for the retrieval, respectively. The blue solid and dashed lines indicate the monthly mean values of simulated hourly O_3_ and OMI-time O_3_ (after the screening), respectively. The red line indicates the monthly mean simulated OMI-time 0–3 km O_3_ column amount with AK.

In order to compare the spatial distributions of the simulation (Fig. 1c) and the observation (Fig. 1d), as shown in Fig. 2, the cross sections of the simulated and observed O_3_ at 117.5°E and 35.5°N are compared, in order to cover the areas, where O_3_ enhancement was observed by the satellite retrieval, namely, the North China Plain and populated and industrial regions in Korea and Japan.

The upper panels of Fig. 2 show the monthly mean values of simulated hourly surface O_3_ (blue, solid), O_3_ at the OMI observed time (blue, dashed), and 0–3 km mean O_3_ at the OMI time (red, solid). The surface mean OMI-time O_3_ was significantly larger by more than 30 ppb than the mean of hourly O_3_ over the area with the abundant presence of emission of precursor gases (30–40°N, 110–120°E). This daytime enhancement was smaller for 0–3 km mean O_3_ but still significant: the 0–3 km mean OMI-time O_3_ was larger by 10–20 ppb over the high emission area. On the other hand, over the low emission area or over the ocean, the 0–3 km mean OMI-time O_3_ was even larger than the surface OMI-time O_3_ due to the absence of photochemical production near the surface. In such areas, the daytime O_3_ enhancement was not detected by the satellite and thus no statistical significance was found between the a priori and retrieved O_3_.

The lower panels of Fig. 2 show the monthly mean values of simulated 0–3 km column amount of hourly O_3_ (blue, solid), O_3_ at the OMI time (blue, dashed), and O_3_ at the OMI time with AK (red, solid). The monthly mean OMI-time O_3_ was 1–4 DU larger than the monthly mean of hourly O_3_ over the large emission areas, but the absolute values of monthly simulated OMI-time O_3_ with AK were even lower than the monthly mean of hourly O_3_, due to the reasons presented later with Eq. (1). Still, the important things here are that the simulated OMI-time O_3_ with AK agreed well with the OMI-O_3_ (black, solid) (generally smaller than 0.5 DU over the high emission areas) and that the both simulated and observed O_3_ were significantly larger than the a priori O_3_ (black, dashed) over the high emission areas. The spatial correlation coefficient between the simulated and observed monthly values over the hatched area in the dashed box shown in Fig. 1d was 0.94.

### Sensitivity to emission changes and statistical significance

**Figure 3:**
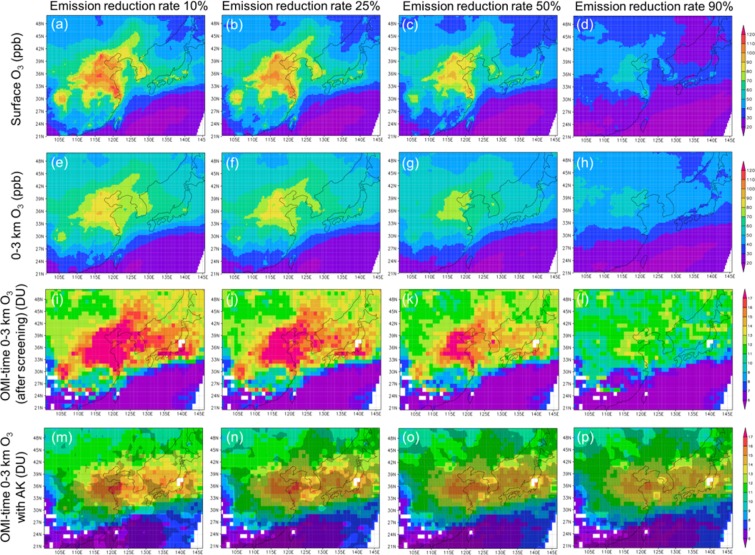
Simulated lower tropospheric O_3_ responses to its precursor emission changes. The monthly mean simulated (top to bottom) surface O_3_ (ppb), 0–3 km mean O_3_ (ppb), OMI-time 0–3 km O_3_ column amount (after the screening) (DU), and OMI-time 0–3 km O_3_ column amount with AK (DU) at the different emission reduction levels of the precursor gases, (left to right), 10%, 25%, 50%, and 90% of the control level. The hatched areas indicate the grids where the simulated O_3_ with AK is significantly smaller than that for the control level of precursor gases emissions at a one-sided 95% confidence interval.

Figure 3 shows the monthly mean simulated (top to bottom) surface O_3_, 0–3 km mean O_3_, OMI-time 0–3 km O_3_ after the screening, and that with AK at the reduction rates of anthropogenic precursor gases emissions of (left to right) 10%, 25%, 50%, and 90%. The contrasts between the results with 10% and 90% reductions become gradually smaller from surface O_3_, 0–3 km O_3_, to OMI-time 0–3 km O_3_ with AK. For the simulated 0–3 km O_3_ with AK, even though the differences looked small, the differences between the emission reduction simulations and the control run (i.e. 0% emission reduction simulation; Fig. 1c) were statistically significant at a one-sided 95% confidence interval (the Student’s *t*-test), as indicated by the hatched areas in Fig. 3m–p. The significance areas became larger as the emission reduction rates were larger. (The same figures without the hatched areas are presented in Fig. S3 to make the results more visible.)

The same horizontal distribution with a different confidence interval, two-sided 99%, is presented in Fig. S4, in the supplement. Similar to the differences between the two confidence intervals as shown in Fig. S2, the hatched areas become smaller with the two-sided 99%, but still cover the high concentration areas such as North China Plain and Yangtze River Delta. The most of the high concentration areas are covered with the hatched areas for the emission reduction rates greater than 25% in Fig. S4f – S4h.

**Figure 4:**
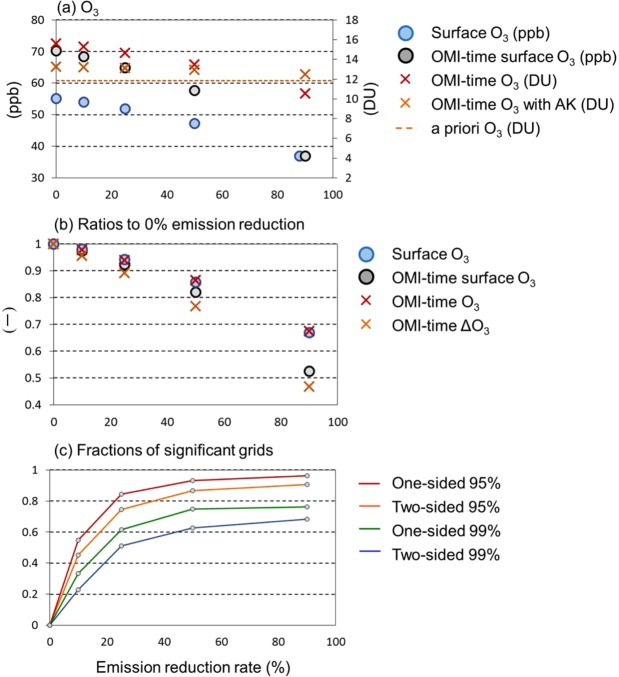
Summary of lower tropospheric O_3_ responses to its precursor emission changes. (**a**) The monthly and areal averaged simulated O_3_ over the areas where the retrieved OMI-O_3_ were significantly larger than the a priori O_3_ (as hatched in Fig. 1d) at the different reduction ratios of the precursor gases emissions (0%, 10%, 25%, 50%, and 90%). (**b**) Same as (**a**) but the ratios to the values at the emission reduction of 0%. (**a**,**b**) The blue and black symbols indicate the mixing ratios of surface hourly O_3_ and OMI-time O_3_ (left axis, ppb), respectively. The red and orange symbols indicate the OMI-time 0–3 km column amounts (after the screening) and that with AK (right axis, DU), respectively. The orange dashed line indicates the areal mean a priori O_3_ column amount. **c**, The blue, green, orange, and red lines indicate the areal fractions at different confidence intervals, two-sided 99%, one-sided 99%, two-sided 95%, and one-sided 95%, respectively.

Figure 4 shows areal statistics of the horizontal distributions over the hatched regions in the dashed box of Fig. 1d: the monthly mean OMI-O_3_ was significantly larger than the a priori O_3_ over 110–146°E and 30–45°N. Some important values in the figure are presented in Table S1. Here we define ΔO_3_. The difference between the OMI-O_3_ and the a priori O_3_ is denoted as OMI-ΔO_3_, which can be regarded as a satellite-detectable O_3_. Also derived and discussed is the simulated ΔO_3_ (or simply referred to as ΔO_3_), which is the simulated O_3_ with AK minus the a priori O_3_ used for the retrieval. ΔO_3_ can be regarded as a simulated satellite-detectable O_3._

Figure 4a,b show the areal and monthly mean simulated O_3_ and their ratios to those at 0% emission reduction, respectively. The black and blue symbols indicate the means of hourly and OMI-time surface O_3_, respectively. The daytime enhancement (black minus blue) was 15 ppb at 0% emission reduction due to the local photochemical productions, which was almost 0 ppb at 90% due to the absence of anthropogenic emissions of precursor gases. O_3_ level at 90% reduction rates can be almost regarded as the background level (or the contributions from the hemispheric transport^11^), approximately 35 ppb for surface O_3_ and 10 DU for 0–3 km column of O_3_. As shown in Fig. 4b, the decreasing rate of the OMI-time surface O_3_ (approximately 50% at 90% reduction) is larger than that of hourly surface O_3_ (approximately 70% at 90% reduction). The 0–3 km OMI-time column amount (red) decreased by 2.3, 6.1, 14, and 33% (Fig. 4b, Table S1) due to the emission reductions of 10, 25, 50, and 90%, respectively. This decreasing trend of the 0–3 km OMI-time column amount (red) was smaller than that of surface OMI-time concentration (black) and as small as that of the surface hourly concentration (blue). The decreasing trend of OMI-time O_3_ with AK (orange in Fig. 4a) is very small, but that of simulated ΔO_3_ (orange in Fig. 4b), i.e., OMI with AK (orange cross) minus the a priori OMI (orange dashed; 11.83 DU), is as large as surface OMI-time O_3_ (Fig. 4b): The ΔO_3_ was reduced to 47% at 90% reduction (Table S1). Therefore, even though the decreasing trend of O_3_ with AK was small, the simulated O_3_ with AK of various emission reduction simulations were significantly smaller than that at 0% reduction simulation, in substantial fractions of the domain (Fig. 4c). The fractions of significant areas at the emission reduction rates of 10%, 25%, 50%, and 90% to the hatched area in Fig. 1d are 55, 84, 93, and 96% at a one-sided 95% confidence interval (red) and 23%, 51%, 63%, and 68% at a two-sided 99% confidence interval (blue), respectively (Fig. 4c).

By using a recently developed satellite product^12,13^ and a regional meteorology – chemistry model^18^, we concluded that the Ozone Monitoring Instrument (OMI) sensor can detect summer-time lower tropospheric O_3_ responses due to reductions of emissions of anthropogenic precursor gases, NO_x_ and non-methane volatile organic compounds (NMVOCs), in East Asia, despite the abundant presence of stratospheric and upper tropospheric O_3_. A socio-economic future emission scenario study^22^ estimated approximately 50% reduction in the global NO_x_ emission in 2100 with compared to the year 2000, and even higher reduction rates up to 80% for additional climate mitigation cases. For such reduction cases greater than 50%, the satellite sensor could detect lower tropospheric O_3_ changes over substantially large areas (Fig. 4c). The current study showed usefulness and importance of monitoring future O_3_ trend by satellite sensors. The same reduction rates between NOx and NMVOC emissions are unlikely in reality, as the emission sources of the two components are very different. The realistic emission scenario needs to be applied in the future beyond the current study. Recently, TROPOspheric Monitoring Instrument (TROPOMI^23^) has been onboard to the Copernicus Sentinel-5 Precursor (S5p) satellite. Because of the recent advancement of satellite sensor technologies, study on tropospheric photochemistry will be rapidly advanced in the near future, together with other technologies such as *in-situ*/remote measurements and numerical simulations.

## Methods

### OMI-retrieved lower tropospheric O_3_ product

The retrieval methodology and the usage of lower tropospheric O_3_ product are described in detail in Hayashida *et al*.^14,15^. We used the data obtained from the OMI sensor, a Dutch-Finnish-built nadir-viewing UV/visible instrument, carried by the Aura spacecraft of the National Aeronautics and Space Administration (NASA) Earth Observing System (EOS) in a sun-synchronous orbit with an equatorial crossing time of ~13:45 local time. The O_3_ profiles are retrieved by Liu *et al*.^12^ with several modifications described in Kim *et al*.^19^, from the ground upward to approximately 60 km in 24 layers, of which 3–7 layers are in the troposphere. To constrain the retrievals, they used climatological zonal mean O_3_ profiles and standard deviations derived from 15 years of ozonesonde measurements and the Stratospheric Aerosol and Gas Experiment (SAGE) as a priori data^17^, which vary with altitude, month, and latitude. The retrieval was performed at a nadir resolution of 52 km × 48 km by adding 4/8 UV1 (270–310 nm) /UV2 (310–365 nm) pixels. In the current study, we use the Level 3 product gridded to 1° × 1° (latitude × longitude) spatial resolution on a daily basis. The gridded data were screened using the effective cloud fraction (ECF) < 0.2 and RMS (root mean square of the ratio of the fitting residual to the assumed measurement error of the UV2 channel) < 2.4 criteria^13^. The retrieved O_3_ in the lower tropospheric layers was found to be affected by outstanding O_3_ enhancement along with the sub-tropical jet due to the intrusion of the stratospheric O_3_^13^. This artifact was successfully screened out by the method developed by Hayashida *et al*.^13,14^. In order to validate their product, Hayashida *et al*.^13^ compared OMI-O_3_ against the *in-situ* airborne measurements, the Measurement of Ozone and Water Vapor by Airbus In-Service Aircraft (MOZAIC) program. Currently, it has been renamed to the Integration of Routine Aircraft Measurements into a Global Observing System (IAGOS) and the data is available at http://www.iagos.fr, last access: 2 November 2019). They found statistically-significant positive correlation for the 0–3 km OMI-O_3_ and that of IAGOS at Beijing from 2004 to 2005, as shown in Table 1.

**Table 1:** Statistical analysis for comparing OMI, NHM-Chem, and the *in-situ* measurements for 0–3 km O_3_.

Product to be evaluated (*y*)	OMI^a^	NHM-Chem^b^	NHM-Chem^c^
*In-situ* measurement (*x*)	IAGOS^a^	IAGOS^b^	ozonesonde^c^
Number of data	36	65–309	80–94
Unit	DU	ppb	ppb
Linear regression	*y* = 0.33*x* + 6.72	—^d^	—^d^
*R*	0.82	0.49–0.72	0.54–0.82
Observed average ($\bar{x}$)	n.a.	38.2–55.8	34.0–45.9
Mean bias ($\bar{y}-\bar{x}$)	n.a.	−1.76–5.2	−3.52–7.94

^a^At Beijing airport for 2004–2005 (Hayashida *et al*.^13^).

^b^Ranges from four airports, Hong Kong, Shanghai, Osaka, and Tokyo for 2005–2006 (this study).

^c^Ranges from four stations, Hong Kong, Naha, Tsukuba, and Sapporo for 2005–2006 (this study).

^c^Linear regression was not presented in this study. The mean bias and observed average values were presented instead.

After validation using the other *in situ* and satellite measurements, the 10 year product (October 2004 – December 2014) was published, referred to as the Smithsonian Astrophysical Observatory (SAO) OMI Ozone Profile (OMPROFOZ)^12,19–21^. Currently, the dataset (V0.9.3) with updates (until September 2019) is available at https://avdc.gsfc.nasa.gov/index.php?site=1389025893&id=74 (last access: 4 November 2019). The data used in the study is equivalent to that V0.9.3.

### A regional-scale meteorology – chemistry model

A regional-scale meteorology – chemistry model, NHM-Chem^18^, was used to simulate tropospheric O_3_ over East Asia. Among the three aerosol representation options employed in NHM-Chem, the bulk equilibrium method was selected in this study. The method is computationally efficient but found to be accurate enough for the prediction of mass concentrations^18^.

The simulation settings such as model domain, simulation period, and boundary conditions are the same as Kajino *et al*.^18^ and thus the details are refrained from repeating. The model domain covers East Asia with 200 × 140 horizontal grid cells with a resolution of Δ*x* = 30 km. The number of grid cells of NHM and CTM were 38 (reaching up to 22,055 m M.S.L.) and 40 (reaching up to 18,000 m M.S.L), respectively, with the terrain-following coordinates. The 3-hourly lateral and upper boundary concentrations of O_3_ and its precursors were obtained from the simulation results of the global stratospheric and tropospheric chemistry – climate model (MRI-CCM2^24^). We used REASv2^4^ for the anthropogenic emissions. The simulation was made for the whole year of 2006, but the analysis was conducted for a month, June 2006, when the monthly mean lower-tropospheric OMI-O_3_ over East Asia was highest in the year. In order to synchronize the OMI observation time (13:45 local time) with the hourly model output time, the simulation results at 6 UTC, which is 13:40 local time at the center of model domain (115°E), were used for the comparison.

Because Kajino *et al*.^18^ only provided model evaluation for surface concentrations, the comparison of the simulated and observed 0–3 km mean concentrations were conducted as shown in Table 1. Because the IAGOS measurement at Beijing ended November of 2005 and no data are available for 2006, we compared the simulation results against the other East Asian airports such as Hong Kong, Shanghai, Osaka, and Tokyo for 2006. In order to increase the number of data, we also drove NHM-Chem for 2005 and compared the results against the observation. We also used the ozonesonde data, conducted by the Global Atmospheric Watch (GAW) program of World Meteorological Organization (WMO) for the model evaluation. The ozonesonde data is available at https://woudc.org/data/explore.php (last access: 2 November 2019). As shown in Table 1, the simulation results showed good agreements with the both *in-situ* measurements.

In addition to Kajino *et al*.^18^, we conducted sensitivity simulations of anthropogenic emission of O_3_ precursors such as NO_x_ and non-methane volatile organic compounds (NMVOCs). We have totally five sets of simulations, with 0%, 10%, 25%, 50%, and 90% reductions from the emission flux of the year 2006. The same reduction rates were applied for both NO_x_ and NMVOCs. Note that the same reduction rates were unlikely in reality as the emission sources of the both species are quite different.

In order to compare against the OMI data, the simulation results were spatially allocated to the grids of the OMI products, which were horizontally 1° × 1° (latitude × longitude) with vertically approximately 3 km intervals. Then, we applied the OMI retrieval averaging kernels (AKs) (rows) to the simulation results for the consistent comparison as follows:

1 $${X}_{j}^{^{\prime} }={X}_{a,j}+\mathop{\sum }\limits_{i=1}^{N}A(i,j)[{X}_{t,i}-{X}_{a,i}]$$

where ${X}_{j}^{^{\prime} }$ is the simulated O_3_ column amount (DU) at *j-*th OMI vertical grid, convolved with the retrieval AKs (*A*(*i*, *j*)), denoted as O_3_ “with AK”. *X*_*t,i*_ is the simulated O_3_ column amount at *i-*th OMI vertical grid and *X*_*a*_ is the a priori profile used in the OMI retrievals. We applied Eq. (1) to the simulation results only for *i* = 22, 23, and 24, (i.e., lower than approximately 9 km above ground level, within the troposphere) and we used the OMI-retrieved data for *X*_*t,i*_ above the 21st layer because NHM-Chem is a tropospheric chemistry model.

## Supplementary information

Supplementary Information

## Data Availability

Data used in Figs. 1–4 and Table 1 are available at https://mri-2.mri-jma.go.jp/owncloud/index.php/s/jGm7cHGVXc8Bp9f. Other data are available upon request to the corresponding author (M.K.).

## Code availability

The NHM-Chem source code is available subject to a license agreement with the Japan Meteorological Agency. Further information is available at http://www.mri-jma.go.jp/Dep/ap/nhmchem_model/application.html. Unfortunately, this website is only in Japanese. Thus additionally, the source code, user’s manual, analysis tools, and sets of boundary conditions can be provided upon request to the corresponding author (M.K.).
