# Supplementary material for: Detectability assessment of a satellite sensor for lower tropospheric ozone responses to its precursors emission changes in East Asian summer
Source: Sci Rep. 2019 Dec 23;9:19629. doi: 10.1038/s41598-019-55759-7 (PMC6928065; doi:10.1038/s41598-019-55759-7)
Supplement: Supplementary file 1 — Supplementary Information [file 41598_2019_55759_MOESM1_ESM.docx]

Supplementary information for “Detectability assessment of a satellite sensor for lower tropospheric ozone responses to its precursors emission changes in East Asian summer”

Mizuo Kajino^1,2^, *, Sachiko Hayashida^3^, Tsuyoshi Thomas Sekiyama^1^, Makoto Deushi^1^, Kazuki Ito^4^, and Xiong Liu

^1^ Meteorological Research Institute (MRI), Japan Meteorological Agency (JMA), Tsukuba, Ibaraki 305-0052, Japan

^2^ Faculty of Life and Environmental Sciences, University of Tsukuba, Tsukuba, Ibaraki 305-8572, Japan

^3^ Faculty of Science, Nara Women’s University, Nara 630-8506, Japan

^4^ Graduate School of Life and Environmental Sciences, University of Tsukuba, Tsukuba, Ibaraki 305-8572, Japan

^5^ Harvard-Smithsonian Center for Astrophysics, Cambridge, MA, 02138, USA

^*^ kajino@mri-jma.go.jp

The supplement shows (1) panels to how the simulation results were compared with OMI-O_3_ and how the horizontal distributions were changed according to the averaging procedures (Fig. S1), (2) the horizontal distributions of statistically significant zones for the 0-3km O_3_ column amounts of OMI (Fig. S2), (3) the same as Fig. 3 but without hatched area (Fig. S3), and (4) the horizontal distributions of the simulation with AK at different emission levels (Fig. S4) at a different confidence interval from the main text: two-sided 99%.

(5) Some important values in Fig. 4 are presented in Table S1.


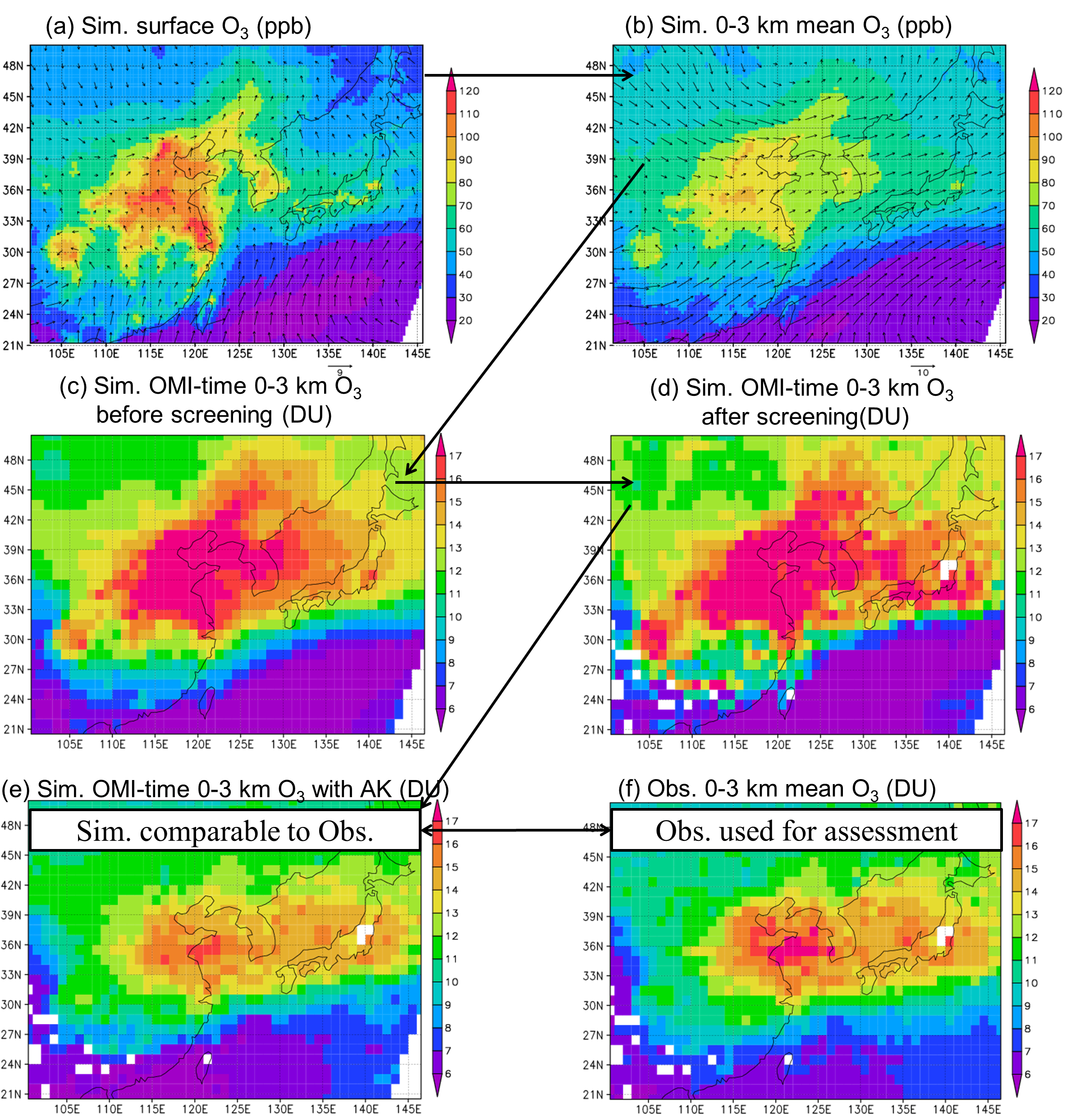


**Figure S1**. (a) Same as Fig. 1 with two additional figures (c) and (d), indicating the simulated OMI-time 0-3 km O_3_ before and after screening for clouds and stratospheric inclusion, respectively. The hatch in Fig. 1d was removed and show in Fig. S1f to make the result more visible. Figs. S1a – S1c are the simulation results to be assessed and Fig. S1e is the converted simulation results, which is quantitatively comparable against the observation, Fig. S1f.

**
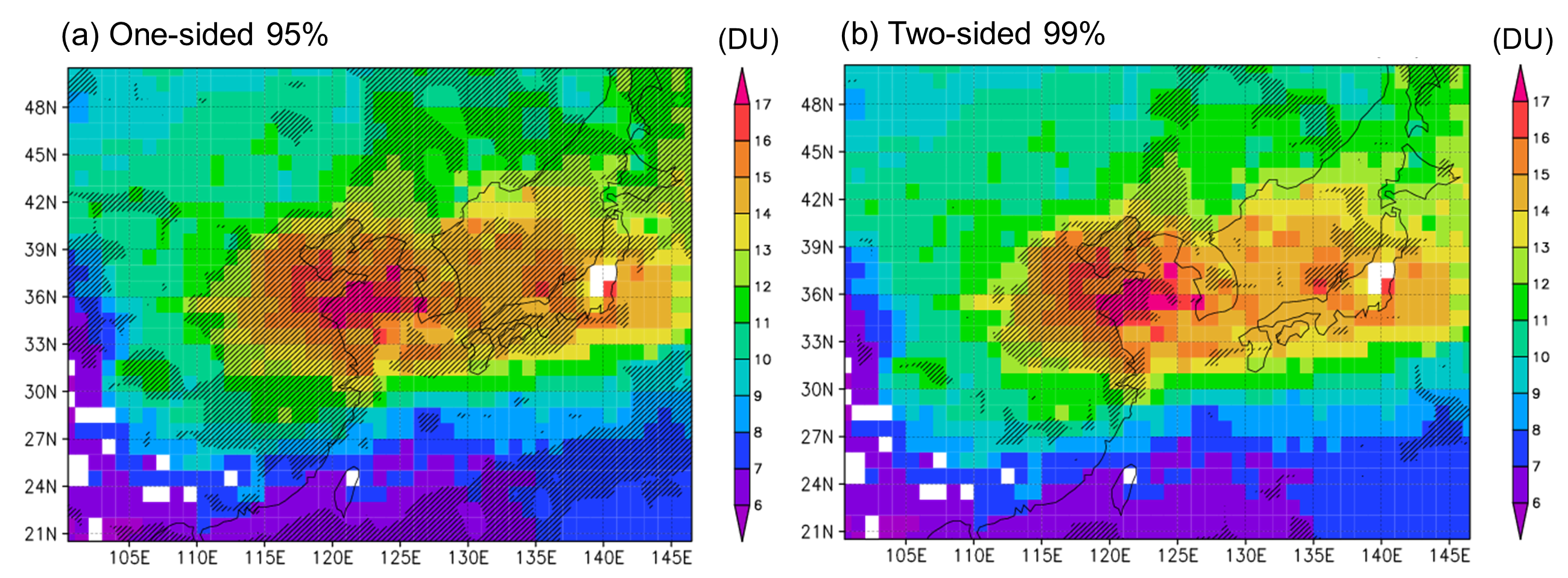
**

**Figure S2**. (a) Same as Fig. 1d. (b) Same as (a) but with the hatched zone at a different confidence interval, two-sided 99%.


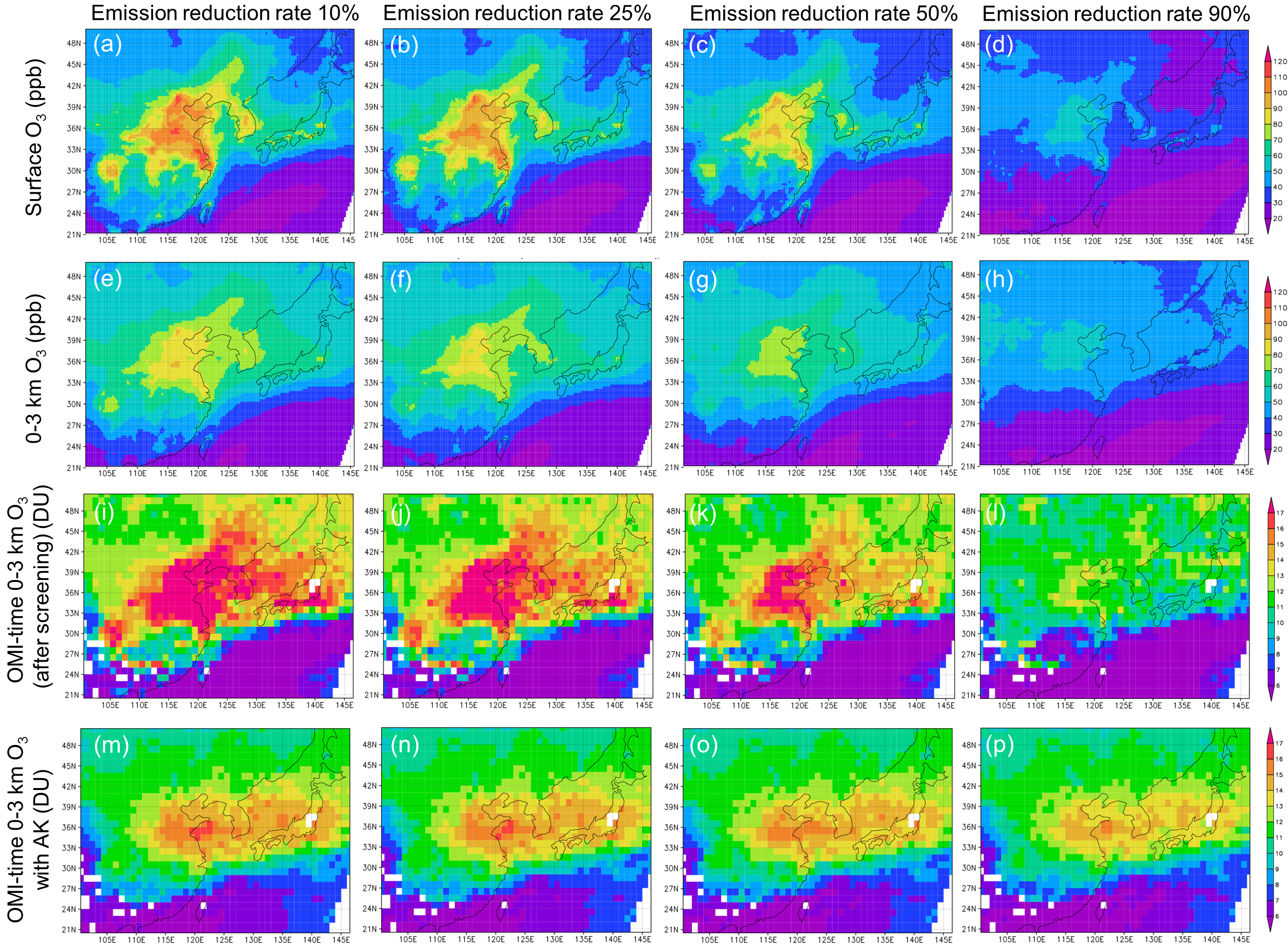


**Figure S3.** Same as Fig. 3 but without hatched areas to make the results more visible.


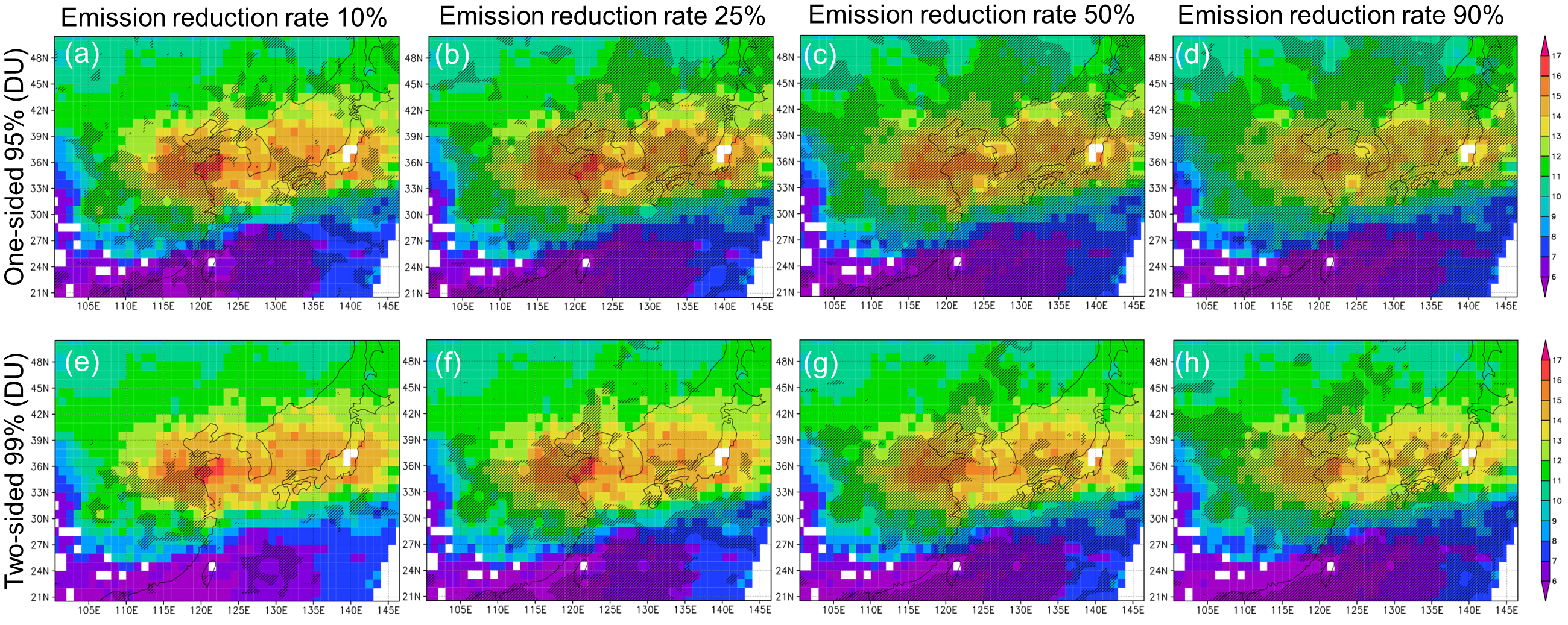


**Figure S4**. (a-d) Same as Fig. 3m-3p. (e-h) Same as (a-d) but with hatched areas at a different confidence interval, two-sided 99%.

**Table S1.** The monthly and areal averaged observed and simulated OMI-time 0-3 km O_3_ column amounts (DU) over the hatched areas in the dashed square regions as shown in Fig. 1d, as presented in Figs. 4a and 4b.

| A priori O_3_ |  |  | 11.83 |
| --- | --- | --- | --- |
| OMI-ΔO_3_ |  |  | 1.659 |
| Simulated | *Emission reduction rate* | O_3_^a^ | ΔO_3_ |
|  | 0% | 15.61 | 1.414 |
|  | 10% | 15.25 (0.98) ^b^ | 1.351 (0.96) |
|  | 25% | 14.66 (0.94) | 1.260 (0.89) |
|  | 50% | 13.49 (0.86) | 1.084 (0.76) |
|  | 90% | 10.53 (0.67) | 0.660 (0.47) |

^a^After screening for clouds and stratospheric intrusion.

^b^Ratios to the 0% emission reduction case are shown in the brackets.
